# High level expression of a glutamate-gated chloride channel gene in reproductive tissues of *Brugia malayi* may explain the sterilizing effect of ivermectin on filarial worms^☆^

**DOI:** 10.1016/j.ijpddr.2014.01.002

**Published:** 2014-01-31

**Authors:** Ben Wen Li, Amy C. Rush, Gary J. Weil

**Affiliations:** Infectious Diseases Division, Department of Internal Medicine, Washington University School of Medicine, St. Louis, MO, USA

**Keywords:** *Brugia malayi*, Glutamate-gated chloride channels, *In situ* hybridization, Ivermectin, Reproduction

## Abstract

Glutamate-gated chloride channels (GluCl) are targets for avermectin/milbemycin (A/M) anthelmintics such as ivermectin that cause paralysis of somatic and pharyngeal muscles in gastrointestinal nematodes. Ivermectin is useful for onchocerciasis control programs because of its activity against microfilariae that often cause ocular disease and severe dermatitis. However, mechanisms responsible for reduced microfilaria production by adult worms following ivermectin treatment are poorly understood. We synthesized subunit-specific RNA probes for the *Brugia malayi* GluCl gene *avr*-14 (*BmAVR*-*14*) to localize expression of this gene in adult filarial worms. Both subunits of *BmAVR*-*14* exhibited very similar expression patterns. In female worms, strong expression signals were detected in the ovary, developing embryos and lateral hypodermal chords, with moderate expression in the uterus wall adjacent to stretched microfilariae. These genes were also highly expressed in adult male worms (in spermatogonia, in the wall of the *vas deferens*, and in the lateral chords, but not in mature spermatozoa). In addition, *avr*-*14* was highly expressed in somatic muscles adjacent to the terminal end of the *vas deferens* which contains mature sperm. These results show that *avr*-*14* is highly expressed in *B. malayi* developing embryos and reproductive tissues, and they provide evidence for the involvement of GluCl in gamete production and embryogenesis in filarial worms. This may explain the observed suppression of microfilaria (Mf) production by female worms following treatment with avermectin/milbemycin anthelmintics.

## Highlights

• Glutamate-gated chloride channels (GluCl) are targets for avermectin/milbemycin (A/M) anthelmintics.
• Little is known regarding the mechanism of A/M anthelmintics to reduce microfilaria production and release in filarial worms.
• We show that two GluCl subunits gene are expressed in developing embryos, and reproductive tissues of adult worms.
• The results may explain the temporary sterilizing effects of A/M drugs on filarial worms.

## Graphical abstract

**Figure:**
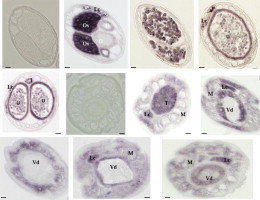

## 1 Introduction

Parasitic nematodes cause major diseases in humans, livestock, and crops (Broughan and Wall, 2007; Coles, 2001; Chitwood, 2003; Hotez et al., 2007). Nematodes are responsible for several important neglected tropical diseases of humans including lymphatic filariasis, onchocerciasis, and soil transmitted helminthiasis (STH) which affect a large portion of the world’s population (Hotez et al., 2007). Because no effective vaccines are available for these infections, anthelmintic drugs are the most important tool available for controlling diseases caused by nematodes (Geary et al., 2010). For example, macrocyclic lactones (MLs) such as ivermectin and moxidectin are routinely used to control nematode infections in animals, and hundreds of millions of people receive ivermectin (IVM) each year in mass drug administration programs for lymphatic filariasis and onchocerciasis (Alleman et al., 2006; Campbell, 2012; Fox, 2006; Martin et al., 2005; Ottesen et al., 2008; Wolstenholme and Rogers, 2005). Ivermectin interferes with neurotransmission in nematode worms by interacting with glutamate-gated chloride channels (GluCls) (Cully et al., 1994). Ivermectin irreversibly activates GluCl in invertebrates (Cully et al., 1994; Forrester et al., 2003; McCavera et al., 2009; Vassilatis et al., 1997a; Yates and Wolstenholme, 2004). In the free-living model nematode *Caenorhabditis elegans*, IVM activates alpha subunits of GluCl channels that are encoded by *avr*-*14*, *avr*-*15*, *glc*-*1*, and *glc*-*3* genes (Dent et al., 1997, 2000; Horoszok et al., 2001). GluCl are members of the Cys-loop ligand-gated ion channel family. While they are only present in invertebrates (Cleland, 1996), they are distantly related to vertebrate γ-aminobutyric acid-A (GABA_A_) receptors (Vassilatis et al., 1997b). Six GluCl genes have been identified in *C. elegans* that encode as many as eight protein subunits that are formed by alternative splicing (Cully et al., 1994; Dent et al., 1997, 2000; Horoszok et al., 2001; Vassilatis et al., 1997b). The *avr*-*14* gene is highly conserved in free-living and parasitic nematodes (Dent et al., 2000; El-Abdellati et al., 2011; Jagannathan et al., 1999; Laughton et al., 1997; McCavera et al., 2007; Njue and Prichard, 2004; Tandon et al., 2006; Williamson et al., 2007; Yates and Wolstenholme, 2004). The gene is alternatively spliced in most species to yield two subunits, GluClα3A and α3B. These subunits share a common N-terminal ligand-binding domain, but they have different C-terminal channel-forming domains. Detailed functional studies of GluCl genes have not been performed for *Brugia malayi,* however, an analysis of the genome shows the presence of genes that encode four GluCl subunits including *BmAVR*-*14A* and *BmAVR*-*14B* (Ghedin et al., 2007; Williamson et al., 2007).

Localization of GluCl gene expression may provide clues regarding the function(s) of these genes in nematode worms. Prior studies of free-living *C. elegans* and gastrointestinal nematode parasites showed that GluCl are expressed in pharyngeal muscle and in motor neurons (Dent et al., 1997, 2000; Gill et al., 1991; Geary et al., 1993; Glendinning et al., 2011; Laughton et al., 1997; Martin, 1996; Portillo et al., 2003). This finding is consistent with the inhibitory effects of avermectin/milbemycin (A/M) anthelmintics on pharyngeal pumping and motor activity in these species. However, other effects of these drugs are not explained by these localization studies. For example, the A/M anthelmintics are effective for treatment of onchocerciasis, because they clear microfilariae (Mf) from the skin and temporarily sterilize adult *Onchocerca volvulus* females (Awadzi et al., 1985). A/M drugs dramatically reduce the release of new Mf by adult worm *in vivo* (Cartel et al., 1993; Klager et al., 1993; Lok et al., 1995; Schares et al., 1994; Stolk et al., 2005) and *in vitro* (Tompkins et al., 2010). They also interfere with embryogenesis in adult female filarial worms (Breton et al., 1997; Chavasse et al., 1992; Duke et al., 1990, 1991, 1992; Lok et al., 1988; Mancebo et al., 1997; Petersen et al., 1996). Ivermectin rapidly reduces Mf in skin or blood (Brown et al., 2000; Basanez et al., 2008; Mak et al., 1993), although the speed and duration of Mf clearance varies for different parasite species. This is likely to be due to direct effects of ivermectin on the Mf nervous system. However, a recent study reported that GluCl was expressed in muscle controlling the excretory/secretory pore of *B. malayi* Mf, and the authors suggested that IVM may affect Mf by interfering with the function of this pore (Moreno et al., 2010). However, these prior studies have not adequately explained the effects of A/M on Mf production and embryogenesis in adult filarial worms. Therefore, the purpose of this study was to assess the expression of GluCl in adult filarial worms and to determine whether GluCl genes are expressed in filarial reproductive tissues. Our results show that this is indeed the case. These results suggest that the effects of A/M on Mf production are not simply due to effects of these drugs on intrauterine Mf.

## 2 Materials and methods

### 2.1 Parasite material and slide preparation

Adult *B. malayi worms were isolated from* infected jirds and separated carefully by gender as previously described (Li et al., 2004). Live worms were washed twice using phosphate buffered saline (PBS) and immediately fixed in 4% formalin buffer. Fixed worms were embedded in paraffin in the Histology Core Laboratory at Washington University School of Medicine. The embedded worms were cut into 5 μm sections, using a microtome. Sections were floated onto Superfrost/Plus microscope slides (Fisher Scientific, Pittsburgh, PA, USA) and placed on a warming block in at 65 °C for 20 min to bond the tissue to the glass. Slides were then stored at room temperature for future use.

### 2.2 Selection of target genes and primer design

Subunit-specific RNA probes were designed with consensus cDNA sequences for *B. malayi avr*-*14* GluCl subunits *BmAVR*-*14A* and *BmAVR*-*14B*. The sequences were retrieved from the NCBI database (http://www.ncbi.nlm.nih.gov/) using accession numbers HQ123446 and HQ123447 (Moreno et al., 2010). Primers were designed using PrimerQuest software (http://idtdna.com/primerquest/home). The primers listed were purchased from Integrated DNA Technology Inc. (Coralville, IA USA). The forward and reverse primers for the two subunits follow: *BmAVR*-*14A*: 5′-AAGGATTCGGTACCTGCTCGTGTT-3′ (forward) and 5′-AACCCAGGAAACGACGACCAACAT-3′ (reverse) with a 419bp amplicon; *BmAVR*-*14B*: 5′-GGTGGTCCAGTGCTAGTCTCTGTAAA-3′ (forward) and 5′-AAGGCATGTTTGTCGATCCAACGG-3′ (reverse) with a 390bp amplicon.

### 2.3 RNA probe construction and *in situ* hybridization

Target gene sequences were amplified by PCR using *B. malayi* adult cDNA template as previously reported (Li et al., 2004). Amplified fragments of the selected genes were cloned into a dual promoter PCRII vector (K2060-0, Invitrogen, Carlsbad, CA, USA) according to the manufacturer’s protocol, and insertion of the fragments was confirmed by sequencing. Biotinlyated anti-sense and sense probes were prepared by reverse transcription from the template plasmid using MEGAscript T7 and Sp6 *in vitro* transcription kits (Ambion, Grand Island, NY, USA) with biotinylated NTPs (Roche Diagnostics, Indianapolis, IN, USA). The biotinylated RNA probes were purified and concentrated by ethanol precipitation, dissolved in DEPC-water, and stored at −20 °C until use.

Paraffin sections were deparaffinized and digested with pepsin HCL for approximately 4 min. Sections were pre-hybridized with hybridization buffer (KPL, catalog #50-86-10, Gaithersburg, Maryland, USA) for 30 min at 37 °C. The sections were hybridized at 60 °C or 42 °C (depending on the probe being used) overnight in a humid chamber with 1 μg/mL of RNA probe in hybridization buffer. An *in situ* hybridization detection system kit (K0601, Dako, Carpinteria, CA, USA) was used for stringency wash and detection. Briefly, sections were washed at 60 °C for 30 min and incubated for 40 min with bioyinylated rRNA with streptavidin-AP conjugate at room temperature. After washing, sections were developed with BCIP/NBT substrate solution for 10–30 min. Slides were viewed using an Olympus-BX40 microscope (Olympus, Tokyo, Japan) and photographed with an Infinity2 digital microscope camera using Infinity Capture software (Lumenera, Ottawa, Ontario, Canada). The signal intensity of each object was scored as strong, moderate or weak according to the intensity of staining to provide a semi-quantitative assessment of gene expression. The stage of the embryos was defined as previously reported (Jiang et al., 2008). According to Lok et al. (1988) the embryonic stages were classified as follows: prelarvae (forms ranging from unfertilised eggs to morulae); developed embryos (forms ranging from morulae with a first invagination to elongated embryos with the two extremities in contact); pretzels (forms ranging from embryos with overlapping extremities to microfilariae coiled within the egg membrane; stretched microfilariae). The distal part of uterus contains mostly prelarval stages, the middle part of uterus mainly contains developing embryos, and the proximal uterus contains pretzel larvae and mature stretched microfilariae (Breton et al., 1997).

## 3 Results and discussion

The localization of *avr*-*14* expression and GluCl proteins has attracted considerable attention, because the protein product of *avr*-*14* is the site of action of A/M anthelmintics (Cully et al., 1994). Prior studies have used reporter gene constructs that could not distinguish between the two splice variants in *C. elegans* and subunit-specific antibodies in *Haemonchus contortus* (Dent et al., 2000; Jagannathan et al., 1999; Portillo et al., 2003). In these species, *avr*-14 gene expression (or Avr-14 protein) were detected in extrapharyngeal neurons in the head, sensory neurons, and ventral cord motor neurons in adults using whole-mount preparations of adult worms. These expression patterns correlated well with the observed action of A/M in these species including inhibition of pharyngeal pumping and spastic muscle contraction. A recent study used an anti-peptide antibody (not subunit-specific) to show that *BmAVR*-*14* was expressed in a muscle structure that surrounds the Mf excretory–secretory (ES) vesicle (Moreno et al., 2010).

**Fig. 1:**
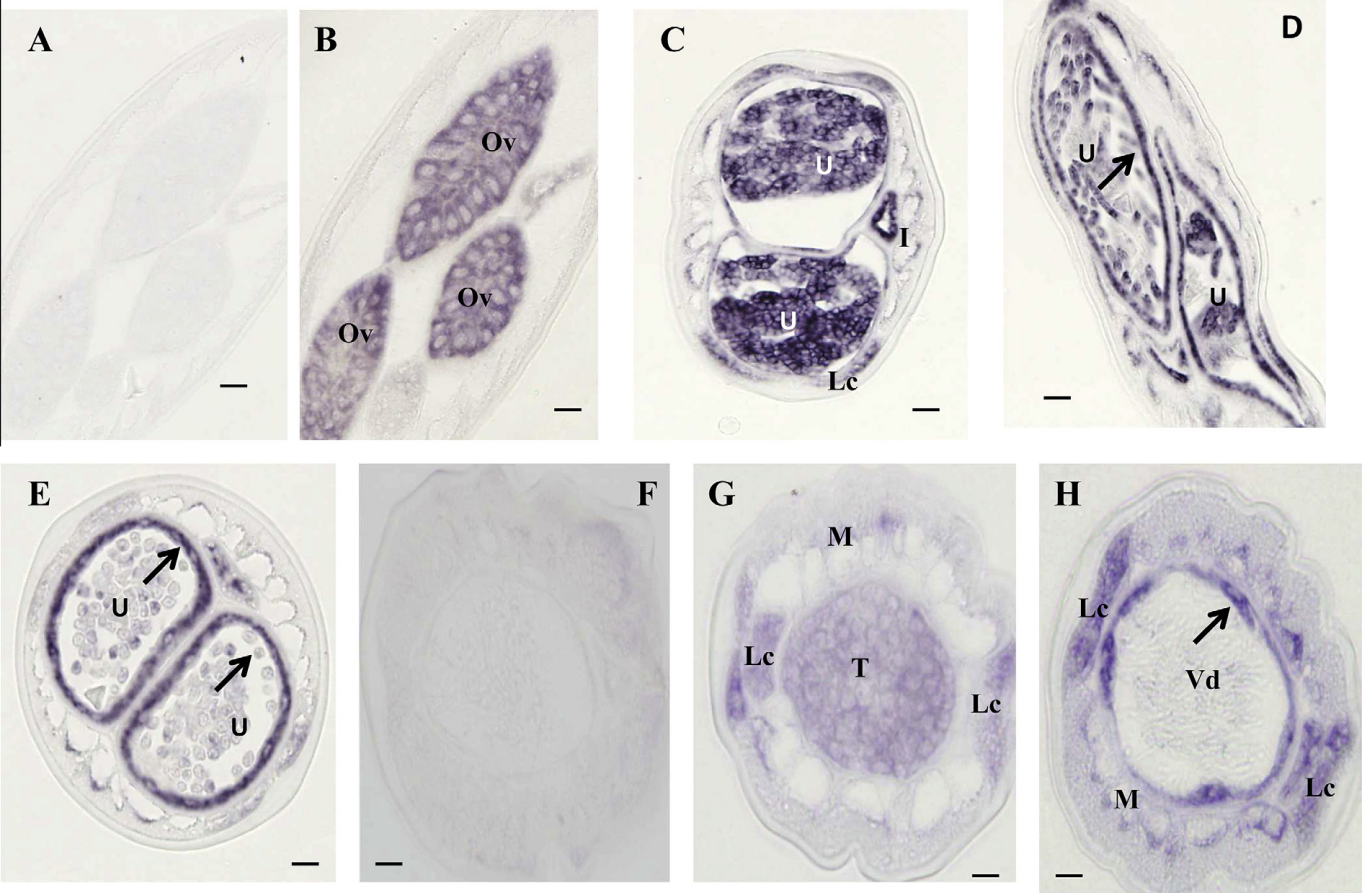
*In situ* hybridization patterns for *BmAVR-14A* in adult *B. malayi* adult worms. The sense RNA probe (negative control) did not label tissues in female (A) or male worms (F). In contrast, the antisense probe produced strong signals in female (B–E) and male worms (G–H). Oocytes in ovary (B), morulae stage embryos (C) and the uterine wall (arrow) adjacent to pretzel or stretched microfilariae (Mf) were intensely labeled (arrows) and stretched Mf were weakly labeled (D–E). The antisense probe also labeled spermatogonia in the male testis (G), the lateral chord (G–H) and the wall of the *vas deferens* (arrow) (H), whereas mature sperm within the *vas deferens* were not labeled (H). Weak to moderate labeling was also observed in the male body wall. Abbreviations: Ov, ovary; I, intestine; U, uterus; M, muscle; Lc, lateral chord, Vd, *vas deferens*; T, testis. Scale bar is 10 μm in panel A–E and 5 μm in panel F–H.

**Fig. 2:**
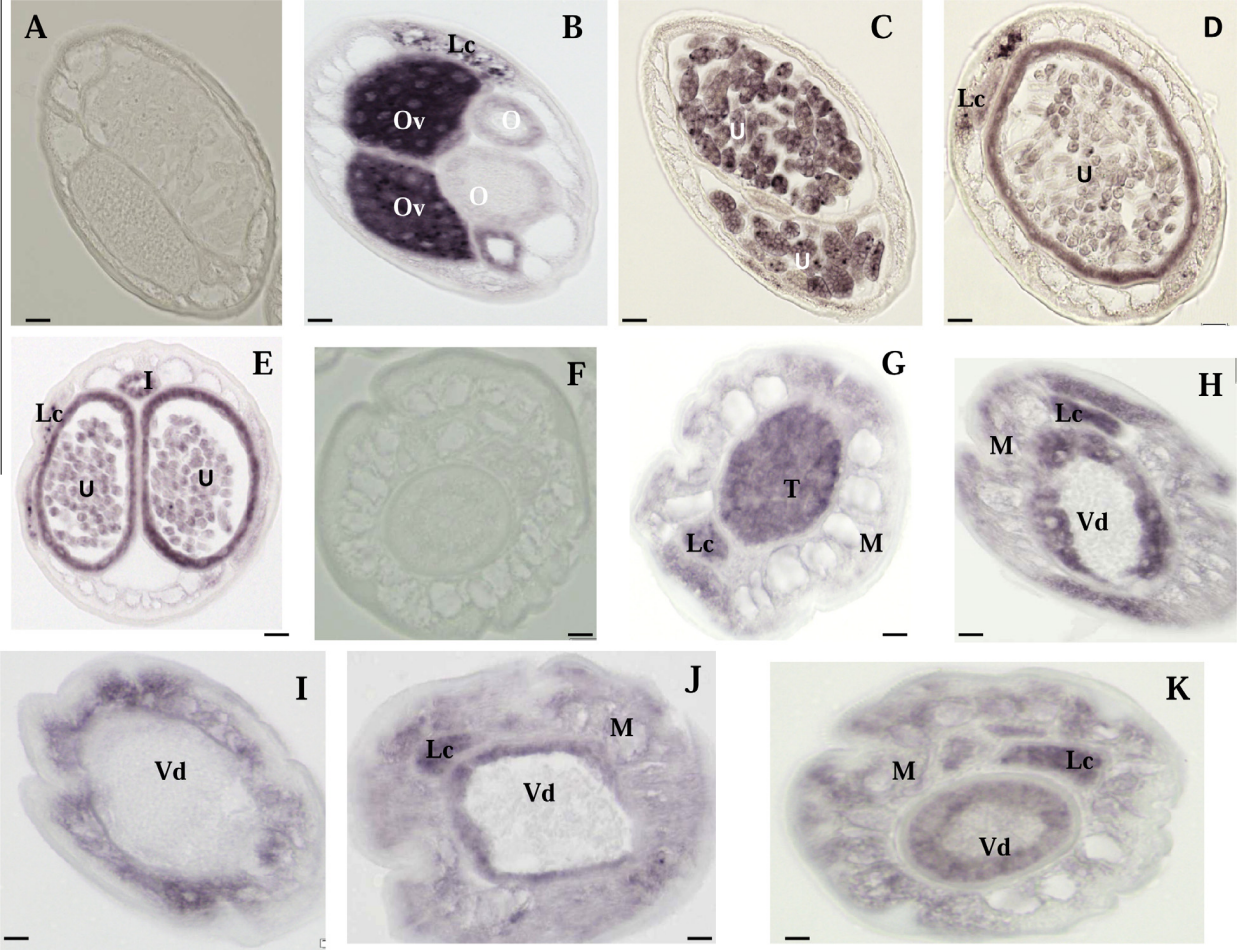
*In situ* hybridization patterns of *BmAVR-14B* in *B. malayi* adult worms. The sense RNA probe (control) did not produce hybridization signals in female or males worms (panels A and F). In contrast, the antisense probe produced strong signals in female (B–E) and male worms (G–K). Oocytes in the ovary (B), morulae (C) and pretzel stage (D) embryos, and the uterine wall adjacent to pretzel or stretched microfilariae (D–E) were intensely labeled; stretched microfilariae were weakly labeled (E). No signal was observed in oviduct. (B) The antisense probe also labeled spermatogonia in the male testis (G), the lateral chord (G–H) and the wall of *vas deferens* (Vd), whereas mature sperm within the Vd were not labeled (H–K). Weak to moderate labeling was observed in the male body wall (H–K). Abbreviations: O, oviduct; Ov, ovary; I, intestine; U, uterus; M, muscle; Lc, lateral chord, Vd, *vas deferens*; T, testis. Scale bar is 10 μm in panel A–E and 5 μm in panel F–K.

We studied expression patterns of *BmAVR*-*14* in adult worms to improve understanding of how IVM affects Mf reproduction and release. We searched for the region of greatest sequence diversity between the two subunits (*BmAVR*-*14A and* -*14B*) and synthesized subunit-specific probes for hybridization studies with sections from adult worms. Before reviewing our results with these probes, we should mention that control probes with sense sequences did not produce hybridization signals in sections from male or female worms (Figs. 1A and F and 2A and F).

Gene expression results for *BmAVR*-*14* are summarized in Table 1. The probes against both subunits produced very similar signals in female worms (Figs. 1B–E and 2B–E). Intense labeling for both transcripts was seen in oocytes in the ovaries (Figs. 1B and 2B), developing morulae (Figs. 1C and 2C), and early pretzel stage larvae (Figs. 1D and 2D). Stretched Mf in the uterus were weakly labeled (Figs. 1E and 2E). Hybridization signals were also observed in the body wall muscle toward the anterior end of female worms where the uterus contains stretched Mf (Figs. 1E and 2E). Both probes produced expression signals in the lateral chords (Figs. 1B and 2D and E). No signal was observed for *BmAVR*-*14B* in the proximal end of the oviduct (spermatheca) (Fig. 2B).

Strong expression of *Bm AVR*-*14* in early embryos (prelarval stages) suggests that this gene plays an important role in embryogenesis, and it is consistent with the observed suppressive effect of A/M on embryogenesis reported in previous studies (Breton et al., 1997; Lok et al., 1988; Tompkins et al., 2010). Prior studies have also shown that ivermectin has a profound effect on embryonic development in *O. volvulus* (Chavasse et al., 1992) and in *Dirofilaria immitis* (Lok et al., 1988). Tompkins, et al observed degenerating embryos/Mf in the uterus of A/M-treated filarial worms (Tompkins et al., 2010). Our findings provide a molecular explanation for the effect of A/M on embryo development and Mf production. The strong expression of *AVR*-*14* in the wall of the uterus with stretched Mf may explain the observed increased proportion of stretched Mf in the uterus and reduction in Mf release following A/M treatment in filarial worms (Lok et al., 1988; Tompkins et al., 2010).

Hybridization results obtained with male worms are summarized in Table 1. Moderate expression signals were observed in spermatogonia in the testis (Figs. 1G and 2G), and strong signals were observed in the lateral chords (Figs. 1G–H and 2G–H) and in the walls of the *vas deferens* which contain spermatozoa, which were not labeled (Figs. 1H and 2H–K). The expression signals of *BmAVR*-*14* increased toward the caudal end of male worms where the somatic muscle and the wall of *vas deferens* are thicker. This was more evident for *BmAVR*-*14B* (Fig. 2I–J). These results suggest that the products of *BmAVR*-*14* may influence body movement, development and release of sperm. Studies of *D. immitis* support this hypothesis. Ivermectin-sterilized *D. immitis* females recovered their ability to produce larvae if they were transferred into normal dogs together with untreated male worms; fertility was not restored if treated females were transferred with treated males (Lok et al., 1988). Embryogenesis was arrested at the single-cell stage possibly because of reduced fertilization as shown by the absence of sperm in the seminal receptacle of female *O. volvulus* following multiple doses of ivermectin, despite the fact that there was no significant reduction in the number of live male worms per nodule (Chavasse et al., 1993). Explantions suggested for this included abnormal spermatogenesis, a disinclination of the male worms to mate, male immobilization due to drug exposure, or a block to the passage of sperm by degenerating Mf in the uteri (Awadzi et al., 1999).Because a prior study showed that the HcGluClα3 subunits A and B in *H. contortus* (protein products of the GluCl gene *avr*-*14*) were expressed in different neurons using subunit-specific antibodies (Portillo et al., 2003), we designed subunit-specific probes for *BmAVR*-*14A* and -*B* and compared their expression patterns in adult *B. malayi* worms. However, both subunits of *BmAVR*-*14* had very similar expression patterns in male and female worms.

In summary, we have used *in situ* hybridization to show that GluCl genes are highly expressed in reproductive tissues of *B. malayi*. These results are novel, because GluCl transcripts or proteins have not been reported in the reproductive organs of other classes of nematodes. Our results suggest that GluCl could be involved in the development of embryos and sperm in adult filarial worms. This may explain the suppressive effects of A/M anthelmintics on Mf production and release in filarial nematodes.

## Figures and Tables

**Table 1:** *BmAVR-14* expression patterns by *in situ* hybridization.^a^

subunit	Female reproductive system								lateral chord	Male reproductive system				
	Uteral- epithelium	Oocytes early	Oocytes later	Morulae early	Morulae later	Pretzel early	Pretzel later	Stretched MF		Spermatogonia	Spermatocytes	Spermatids	Spermatozoa	Vas deferens
BmAVR-14A	3	3	3	2	2	1	1	1	1	2	1	0	0	2
BmAVR-14B	3	3	3	2	2	1	2	1	2	3	1	0	0	2

a Signal intensity was scored as follows: 1, weak; 2, moderate; 3, strong.

## References

[b0015] Awadzi K., Dadzie K.Y., Shulz-Key H., Haddock D.R., Gilles H.M., Aziz M.A. (1985). The chemotherapy of onchocerciasis X. An assessment of four single dose treatment regimes of MK-933 (ivermectin) in human onchocerciasis. Ann. Trop. Med. Parasitol..

[b0010] Awadzi K., Attah S.K., Addy E.T., Opoku N.O., Quartey B.T.Q. (1999). The effects of high-dose ivermectin regimens on *Onchocerca volvulus* in onchocerciasis patients. Trans. R. Soc. Trop. Med. Hyg..

[b0005] Alleman M.M., Twum-Danso N.A., Thylefors B.I. (2006). The Mectizan donation program – highlights from 2005. Filaria J..

[b0025] Breton B., Diagne M., Wanji S., Bougnoux M.E., Chandre F., Marechal P., Petit G., Vuong P.N., Bain O. (1997). Ivermectin and moxidectin in two filarial systems: resistance of Monanema martini; inhibition of *Litomosoides sigmodontis* insemination. Parassitologia.

[b0035] Brown K.R., Ricci F.M., Ottesen E.A. (2000). Ivermectin: effectiveness in lymphatic filariasis. Parasitology.

[b0030] Broughan J.M., Wall R. (2007). Faecal soiling and gastrointestinal helminth infection in lambs. Int. J. Parasitol..

[b0020] Basanez M.G., Pion S.D., Boakes E., Filipe J.A., Churcher T.S., Boussinesq M. (2008). Effect of single-dose ivermectin on *Onchocerca volvulus*: a systematic review and meta-analysis. Lancet Infect. Dis..

[b0050] Chavasse D.C., Post R.J., Lemoh P.A., Whitworth J.A. (1992). The effect of repeated doses of ivermectin on adult female *Onchocerca volvulus* in Sierra Leone. Trop. Med. Parasitol..

[b0045] Cartel J.L., Moulia-Pelat J.P., Glaziou P., Nguyen L.N., Chanteau S., Roux J.F., Spiegel A. (1993). Microfilariae recurrence in Polynesian *Wuchereria bancrofti* carriers treated with repeated single doses of 100 micrograms/kg of ivermectin. Trans. R. Soc. Trop. Med. Hyg..

[b0055] Chavasse D.C., Post R.J., Lemoh P.A., Whitworth J.A. (1993). Absence of sperm from the seminal receptacle of female *Onchocerca volvulus* following multiple doses of ivermectin. Trop. Med. Parasitol..

[b0075] Cully D.F., Vassilatis D.K., Liu K.K., Paress P.S., Van der Ploeg L.H., Schaeffer J.M., Arena J.P. (1994). Cloning of an avermectin-sensitive glutamate-gated chloride channel from *Caenorhabditis elegans*. Nature.

[b0065] Cleland T.A. (1996). Inhibitory glutamate receptor channels. Mol. Neurobiol..

[b0070] Coles G.C. (2001). The future of veterinary parasitology. Vet. Parasitol..

[b0060] Chitwood D.J. (2003). Research on plant-parasitic nematode biology conducted by the United States department of agriculture–agricultural research service. Pest Manag. Sci..

[b0040] Campbell W.C. (2012). History of avermectin and ivermectin, with notes on the history of other macrocyclic lactone antiparasitic agents. Curr. Pharm. Biotechnol..

[b0090] Duke B.O., Zea-Flores G., Castro J., Cupp E.W., Munoz B. (1990). Effects of multiple monthly doses of ivermectin on adult *Onchocerca volvulus*. Am. J. Trop. Med. Hyg..

[b0100] Duke B.O., Zea-Flores G., Munoz B. (1991). The embryogenesis of *Onchocerca volvulus* over the first year after a single dose of ivermectin. Trop. Med. Parasitol..

[b0095] Duke B.O., Zea-Flores G., Castro J., Cupp E.W., Munoz B. (1992). Effects of three-month doses of ivermectin on adult *Onchocerca volvulus*. Am. J. Trop. Med. Hyg..

[b0080] Dent J.A., Davis M.W., Avery L. (1997). Avr-15 encodes a chloride channel subunit that mediates inhibitory glutamatergic neurotransmission and ivermectin sensitivity in *Caenorhabditis elegans*. Embo J..

[b0085] Dent J.A., Smith M.M., Vassilatis D.K., Avery L. (2000). The genetics of ivermectin resistance in *Caenorhabditis elegans*. Proc. Natl. Acad. Sci. USA.

[b0105] El-Abdellati A., De Graef J., Van Zeveren A., Donnan A., Skuce P., Walsh T., Wolstenholme A., Tait A., Vercruysse J., Claerebout E., Geldhof P. (2011). Altered avr-14B gene transcription patterns in ivermectin-resistant isolates of the cattle parasites, *Cooperia oncophora* and *Ostertagia ostertagi*. Int. J. Parasitol..

[b0110] Forrester S.G., Prichard R.K., Dent J.A., Beech R.N. (2003). *Haemonchus contortus*: HcGluCla expressed in Xenopus oocytes forms a glutamate-gated ion channel that is activated by ibotenate and the antiparasitic drug ivermectin. Mol. Biochem. Parasitol..

[b0115] Fox L.M. (2006). Ivermectin: uses and impact 20 years on. Curr. Opin. Infect. Dis..

[b0135] Gill J.H., Redwin J.M., van Wyk J.A., Lacey E. (1991). Detection of resistance to ivermectin in *Haemonchus contortus*. Int. J. Parasitol..

[b0120] Geary T.G., Sims S.M., Thomas E.M., Vanover L., Davis J.P., Winterrowd C.A., Klein R.D., Ho N.F., Thompson D.P. (1993). *Haemonchus contortus*: ivermectin-induced paralysis of the pharynx. Exp. Parasitol..

[b0130] Ghedin E., Wang S., Spiro D., Caler E., Zhao Q., Crabtree J., Allen J.E., Delcher A.L., Guiliano D.B., Miranda-Saavedra D., Angiuoli S.V., Creasy T., Amedeo P., Haas B., El-Sayed N.M., Wortman J.R., Feldblyum T., Tallon L., Schatz M., Shumway M., Koo H., Salzberg S.L., Schobel S., Pertea M., Pop M., White O., Barton G.J., Carlow C.K., Crawford M.J., Daub J., Dimmic M.W., Estes C.F., Foster J.M., Ganatra M., Gregory W.F., Johnson N.M., Jin J., Komuniecki R., Korf I., Kumar S., Laney S., Li B.W., Li W., Lindblom T.H., Lustigman S., Ma D., Maina C.V., Martin D.M., McCarter J.P., McReynolds L., Mitreva M., Nutman T.B., Parkinson J., Peregrin-Alvarez J.M., Poole C., Ren Q., Saunders L., Sluder A.E., Smith K., Stanke M., Unnasch T.R., Ware J., Wei A.D., Weil G., Williams D.J., Zhang Y., Williams S.A., Fraser-Liggett C., Slatko B., Blaxter M.L., Scott A.L. (2007). Draft genome of the filarial nematode parasite *Brugia malayi*. Science.

[b0125] Geary T.G., Woo K., McCarthy J.S., Mackenzie C.D., Horton J., Prichard R.K., de Silva N.R., Olliaro P.L., Lazdins-Helds J.K., Engels D.A., Bundy D.A. (2010). Unresolved issues in anthelmintic pharmacology for helminthiases of humans. Int. J. Parasitol..

[b0140] Glendinning S.K., Buckingham S.D., Sattelle D.B., Wonnacott S., Wolstenholme A.J. (2011). Glutamate-gated chloride channels of *Haemonchus contortus* restore drug sensitivity to ivermectin resistant *Caenorhabditis elegans*. PLoS One.

[b0145] Horoszok L., Raymond V., Sattelle D.B., Wolstenholme A.J. (2001). GLC-3: a novel fipronil and BIDN-sensitive, but picrotoxinin-insensitive, l-glutamate-gated chloride channel subunit from *Caenorhabditis elegans*. Br. J. Pharmacol..

[b0150] Hotez P.J., Molyneux D.H., Fenwick A., Kumaresan J., Sachs S.E., Sachs J.D., Savioli L. (2007). Control of neglected tropical diseases. N. Engl. J. Med..

[b0155] Jagannathan S., Laughton D.L., Critten C.L., Skinner T.M., Horoszok L., Wolstenholme A.J. (1999). Ligand-gated chloride channel subunits encoded by the *Haemonchus contortus* and *Ascaris suum* orthologues of the *Caenorhabditis elegans gbr-2* (avr-14) gene. Mol. Biochem. Parasitol..

[b0160] Jiang D., Li B.W., Fischer P.U., Weil G.J. (2008). Localization of gender-regulated gene expression in the filarial nematode *Brugia malayi*. Int. J. Parasitol..

[b0165] Klager S., Whitworth J.A., Post R.J., Chavasse D.C., Downham M.D. (1993). How long do the effects of ivermectin on adult *Onchocerca volvulus* persist?. Trop. Med. Parasitol..

[b0180] Lok J.B., Harpaz T., Knight D.H. (1988). Abnormal patterns of embryogenesis in *Dirofilaria immitis* treated with ivermectin. J. Helminthol..

[b0185] Lok J.B., Knight D.H., Selavka C.M., Eynard J., Zhang Y., Bergman R.N. (1995). Studies of reproductive competence in male *Dirofilaria immitis* treated with milbemycin oxime. Trop. Med. Parasitol..

[b0170] Laughton D.L., Lunt G.G., Wolstenholme A.J. (1997). Alternative splicing of a *Caenorhabditis elegans* gene produces two novel inhibitory amino acid receptor subunits with identical ligand binding domains but different ion channels. Gene.

[b0175] Li B.W., Rush A.C., Tan J., Weil G.J. (2004). Quantitative analysis of gender-regulated transcripts in the filarial nematode *Brugia malayi* by real-time RT-PCR. Mol. Biochem. Parasitol..

[b0190] Mak J.W., Navaratnam V., Grewel J.S., Mansor S.M., Ambu S. (1993). Treatment of subperiodic *Brugia malayi* infection with a single dose of ivermectin. Am. J. Trop. Med. Hyg..

[b0200] Martin R.J. (1996). An electrophysiological preparation of *Ascaris suum* pharyngeal muscle reveals a glutamate-gated chloride channel sensitive to the avermectin analogue, milbemycin D. Parasitology.

[b0195] Mancebo O.A., Verdi J.H., Bulman G.M. (1997). Comparative efficacy of moxidectin 2% equine oral gel and ivermectin 2% equine oral paste against *Onchocerca cervicalis* (Railliet and Henry, 1910) microfilariae in horses with naturally acquired infections in Formosa (Argentina). Vet. Parasitol..

[b0205] Martin R.J., Verma S., Levandoski M., Clark C.L., Qian H., Stewart M., Robertson A.P. (2005). Drug resistance and neurotransmitter receptors of nematodes: recent studies on the mode of action of levamisole. Parasitology.

[b0215] McCavera S., Walsh T.K., Wolstenholme A.J. (2007). Nematode ligand-gated chloride channels: an appraisal of their involvement in macrocyclic lactone resistance and prospects for developing molecular markers. Parasitology.

[b0210] McCavera S., Rogers A.T., Yates D.M., Woods D.J., Wolstenholme A.J. (2009). An ivermectin-sensitive glutamate-gated chloride channel from the parasitic nematode *Haemonchus contortus*. Mol. Pharmacol..

[b0220] Moreno Y., Nabhan J.F., Solomon J., Mackenzie C.D., Geary T.G. (2010). Ivermectin disrupts the function of the excretory-secretory apparatus in microfilariae of *Brugia malayi*. Proc. Natl. Acad. Sci. USA.

[b0225] Njue A.I., Prichard R.K. (2004). Genetic variability of glutamate-gated chloride channel genes in ivermectin-susceptible and -resistant strains of *Cooperia oncophora*. Parasitology.

[b0230] Ottesen E.A., Hooper P.J., Bradley M., Biswas G. (2008). The global programme to eliminate lymphatic filariasis: health impact after 8 years. PLoS Negl. Trop. Dis..

[b0235] Petersen M.B., Varady M., Bjorn H., Nansen P. (1996). Efficacies of different doses of ivermectin against male, female and L4 *Oesophagostomum dentatum* in pigs. Vet. Parasitol..

[b0240] Portillo V., Jagannathan S., Wolstenholme A.J. (2003). Distribution of glutamate-gated chloride channel subunits in the parasitic nematode *Haemonchus contortus*. J. Comp. Neurol..

[b0245] Schares G., Hofmann B., Zahner H. (1994). Antifilarial activity of macrocyclic lactones: comparative studies with ivermectin, doramectin, milbemycin A4 oxime, and moxidectin in *Litomosoides carinii*, *Acanthocheilonema viteae*, *Brugia malayi*, and *B. pahangi* infection of Mastomys coucha. Trop. Med. Parasitol..

[b0250] Stolk W.A., VAN Oortmarssen G.J., Pani S.P., DE Vlas S.J., Subramanian S., Das P.K., Habbema J.D. (2005). Effects of ivermectin and diethylcarbamazine on microfilariae and overall microfilaria production in *bancroftian* filariasis. Am. J. Trop. Med. Hyg..

[b0255] Tandon R., LePage K.T., Kaplan R.M. (2006). Cloning and characterization of genes encoding alpha and beta subunits of glutamate-gated chloride channel protein in *Cylicocyclus nassatus*. Mol. Biochem. Parasitol..

[b0260] Tompkins J.B., Stitt L.E., Ardelli B.F. (2010). *Brugia malayi*: in vitro effects of ivermectin and moxidectin on adults and microfilariae. Exp. Parasitol..

[b0265] Vassilatis D.K., Arena J.P., Plasterk R.H., Wilkinson H.A., Schaeffer J.M., Cully D.F., Van der Ploeg L.H. (1997). Genetic and biochemical evidence for a novel avermectin-sensitive chloride channel in *Caenorhabditis elegans.* Isolation and characterization. J. Biol. Chem..

[b0270] Vassilatis D.K., Elliston K.O., Paress P.S., Hamelin M., Arena J.P., Schaeffer J.M., Van der Ploeg L.H., Cully D.F. (1997). Evolutionary relationship of the ligand-gated ion channels and the avermectin-sensitive, glutamate-gated chloride channels. J. Mol. Evol..

[b0280] Wolstenholme A.J., Rogers A.T. (2005). Glutamate-gated chloride channels and the mode of action of the avermectin/milbemycin anthelmintics. Parasitology.

[b0275] Williamson S.M., Walsh T.K., Wolstenholme A.J. (2007). The cys-loop ligand-gated ion channel gene family of *Brugia malayi* and *Trichinella spiralis*: a comparison with *Caenorhabditis elegans*. Invert. Neurosci..

[b0285] Yates D.M., Wolstenholme A.J. (2004). *Dirofilaria immitis*: identification of a novel ligand-gated ion channel-related polypeptide. Exp. Parasitol..

